# Global Genetic Architecture of an Erythroid Quantitative Trait Locus, *HMIP-2*

**DOI:** 10.1111/ahg.12077

**Published:** 2014-07-29

**Authors:** Stephan Menzel, Helen Rooks, Diana Zelenika, Siana N Mtatiro, Akshala Gnanakulasekaran, Emma Drasar, Sharon Cox, Li Liu, Mariam Masood, Nicholas Silver, Chad Garner, Nisha Vasavda, Jo Howard, Julie Makani, Adekunle Adekile, Betty Pace, Tim Spector, Martin Farrall, Mark Lathrop, Swee Lay Thein

**Affiliations:** 1 King's College LondonLondon, UK; 2 Centre National de GénotypageEvry, France; 3 Muhimbili UniversityDar es Salaam, Tanzania; 4 King's College Hospital NHS Foundation TrustLondon, UK; 5 University of Texas at DallasRichardson, TX, USA; 6 University of California Irvine School of MedicineIrvine, CA, USA; 7 Guy's and St Thomas’ Hospital NHS Foundation TrustLondon, UK; 8 Faculty of Medicine, Kuwait UniversityKuwait; 9 Georgia Regents UniversityAugusta, GA, USA; 10 Division of Cardiovascular Medicine, Radcliffe Department of Medicine, Wellcome Trust Centre for Human Genetics, University of OxfordOxford, UK; 11 McGill UniversityMontreal, Canada

**Keywords:** Red blood cells, quantitative trait locus, population genetics, malaria, sickle cell disease, cMYB, gene enhancer variant

## Abstract

*HMIP-2* is a human quantitative trait locus affecting peripheral numbers, size and hemoglobin composition of red blood cells, with a marked effect on the persistence of the fetal form of hemoglobin, HbF, in adults. The locus consists of multiple common variants in an enhancer region for *MYB* (chr 6q23.3), which encodes the hematopoietic transcription factor cMYB. Studying a European population cohort and four African-descended groups of patients with sickle cell anemia, we found that all share a set of two spatially separate HbF-promoting alleles at *HMIP-2*, termed “A” and “B.” These typically occurred together (“A–B”) on European chromosomes, but existed on separate homologous chromosomes in Africans. Using haplotype signatures for “A” and “B,” we interrogated public population datasets. Haplotypes carrying only “A” or “B” were typical for populations in Sub-Saharan Africa. The “A–B” combination was frequent in European, Asian, and Amerindian populations. Both alleles were infrequent in tropical regions, possibly undergoing negative selection by geographical factors, as has been reported for malaria with other hematological traits. We propose that the ascertainment of worldwide distribution patterns for common, HbF-promoting alleles can aid their further genetic characterization, including the investigation of gene–environment interaction during human migration and adaptation.

## Introduction

Human red blood cells have long appealed to geneticists because of their significant contribution to genetic disease, their exceptional accessibility and their relatively simple biology. For decades, genetic studies were focused on Mendelian traits affecting hemoglobin or the erythrocyte membrane. More recently, complex, i.e., quantitative, erythroid traits have become accessible to systematic genetic dissection, leading to the discovery of a large number of common genetic variants influencing red blood cell function and appearance (Sankaran & Orkin, 2013). One such quantitative trait locus (QTL) is *HBS1L-MYB intergenic polymorphism* (*HMIP*) on Chromosome 6q23.3, which was first detected in a large Asian Indian family (“Family D”) of Gujarati/North Indian descent (Thein & Weatherall, 1989; Craig et al., 1996), where it causes autosomal-dominant inheritance of hereditary persistence of fetal hemoglobin (HPFH). Usually, fetal hemoglobin (HbF) production reduces dramatically after birth, when it is replaced by adult hemoglobin (HbA and small amounts of HbA_2_), but some individuals, including members of Family D, continue to produce significant amounts of HbF. When such “HPFH” occurs in patients with sickle cell anemia (SCA; Platt et al., 1994) or β-thalassemia (Ho et al., 1998), where HbA is either defective or diminished, it results in a clinically milder disease. In Family D, the locus alleviates the phenotype of an independently segregating β-thalassemia allele. Subsequently, it was shown that variants at this locus also contribute to a limited, but variable, HbF persistence that exists in the general European population. This finding enabled its subsequent fine-mapping to a 75-kb interval between *HBS1L* and *MYB* and its partitioning into three independent linkage disequilibrium (LD) blocks of common genetic variants associated with the trait (Thein et al., 2007). In Family D, segregation of a single large-effect haplotype at *HBS1L-MYB* is consistent with the observed Mendelian inheritance pattern of HPFH. In this haplotype, the three blocks of associated variants are present in an unusual optimum alignment producing a strong combined effect on the trait. In the general European population, these blocks were found to predominantly exist in different combinations (Thein et al., 2007), leading to the appearance of *HBS1L-MYB* as a more conventional QTL that contributes to the complex genetic determination of HbF persistence.

**Figure 1:**
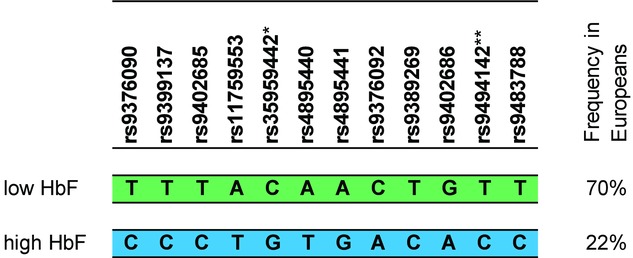
Composition of the two main haplotypes involving HbF-associated variants at *HMIP-2* in healthy Europeans. When investigating the twelve *HMIP-2* SNPs originally reported to be strongly associated with HbF persistence (Thein et al., 2007), we found that very close linkage disequilibrium (LD) between them resulted in two major haplotypes dominating (together 92% of chromosomes) our European cohort. The haplotype shown in green was associated with low HbF (and low F cell) levels, the one below in blue with high levels of both related traits. The high-HbF haplotype is also present at the core of a Chromosome 6 segment segregating with fetal-hemoglobin persistence in the Gujarati Family D, where this locus was first discovered.*previously *rs52090909*; **previously *rs11154792*.

Subsequently, these *HBS1L-MYB* variants have been shown to also modulate HbF levels in healthy subjects of African and East Asian descent and in SCA and β-thalassemia patients and carriers of diverse ethnic origin (Lettre et al., 2008; Gibney et al., 2008; So et al., 2008; Creary et al., 2009; Galanello et al., 2009; Makani et al., 2010; Solovieff et al., 2010; Galarneau et al., 2010; Nuinoon et al., 2010; Farrell et al., 2011; Bae et al., 2012). *HBS1L-MYB* variation has considerable pleiotropic effects, as it also influences the number, size, and overall hemoglobin content of red blood cells (Menzel et al., 2007b; Soranzo et al., 2009b; Kamatani et al., 2010; van der Harst et al., 2012). In addition, it affects circulating numbers of platelets, monocytes, and white cells (Menzel et al., 2007b; Soranzo et al., 2009a; Kamatani et al., 2010; Nalls et al., 2011; Okada et al., 2011; Reiner et al., 2011; Qayyum et al., 2012). Much of the effect of the locus originates from the core block of variants, termed *HMIP-2* (block 2), which occupies a 24-kb stretch of DNA that acts as a distal upstream enhancer for *MYB* (Wahlberg et al., 2009; Stadhouders et al., 2012; Stadhouders et al., 2014), the gene for cMYB, a transcription factor essential to hematopoiesis (Mucenski et al., 1991). *HMIP-2* is one of the most significant and consistently detected loci for erythroid traits across human populations. Noticeably, top-associated SNPs detected in studies performed in European, African, and Asian populations (Creary et al., 2009; Makani et al., 2010) appear to belong to a common set of SNPs, recurring with variation, across studies. This might reflect a shared origin for at least part of the trait-associated variability. In Europeans, a single principal haplotype (frequency 22%), characterized by 12 closely linked SNP alleles distributed over *HMIP-2* (Fig.1), had been shown to be responsible for HbF-increasing effects at *HMIP-2* (Thein et al., 2007). We found the same haplotype prevalent (also at 22% frequency) in the Gujarati population and at the centre of the chromosomal segment segregating with HPFH in Family D (Thein et al., 2007). These findings suggests that a European-type HbF-promoting sequence at *HMIP-2* is an essential part of the extended haplotype (involving HbF-promoting variants of *HMIP-1*, *HMIP-2*, and *HMIP-3*) causing HPFH in this family. Subsets of these 12 SNPs have shown association with erythroid traits in every human population studied so far (Thein et al., 2007; Menzel et al., 2007a, b; Uda et al., 2008; Lettre et al., 2008; Gibney et al., 2008; So et al., 2008; Creary et al., 2009; Galanello et al., 2009; Soranzo et al., 2009b; Ganesh et al., 2009, Nuinoon et al., 2010, Kamatani et al., 2010; Galarneau et al., 2010; Makani et al., 2010; Solovieff et al., 2010; Reiner et al., 2011; Okada et al., 2011; Farrell et al., 2011; Nalls et al., 2011; Qayyum et al., 2012; Bae et al., 2012; van der Harst et al., 2012). In this paper, we describe the *HMIP-2* locus and its characteristic HbF-boosting alleles in a diverse set of human populations. The “HPFH +” haplotype segregating in Family D served as a reference in our investigations, since the strong HbF-boosting effect in all 74 identical-by-descent copies has provided us with a “archetype” of an invariably HbF-promoting sequence across the 24-kb *HMIP-2* interval. We first studied the variants characterizing this sequence in individuals where we have measured HbF persistence: (1) a cohort of healthy European twins and (2) patients of African descent with SCA. Subsequently, we investigated the prevalence of haplotypes signaling the presence of trait-affecting functional variants in human populations across the world, interrogating data from the 1000 Genomes Project (Abecasis et al., 2010) and the Human Genome Diversity Project (HGDP; Pickrell et al., 2009). We provide evidence that most human populations share a set of HbF-inducing haplotypes, which contain two HbF-boosting alleles either separately or in tandem. We discuss the physical location of these alleles at the MYB enhancer, and how they might contribute to the haplotype-specific effects we observe in healthy subjects and patients with SCA in the light of recent functional studies.

## Materials and Methods

### Subjects and Trait Measurement

Subjects were recruited and studied according to the Declaration of Helsinki and gave informed consent. Investigation of the African British patient cohort was approved by the National Research Ethics Service Committee South Central (07/H0606/165) and of the Tanzanian patient cohort by the Muhimbili University Research and Publications Committee (MU/RP/AEC/VOL XI/33). The African American patient cohort is part of a multicentre study (see below), approved by the Institutional Review Boards at the collaborating institutions. The Nigerian patients are part of an archival cohort that was analyzed anonymously. Study of the Twins UK cohort was approved by the St. Thomas’ Research Ethics Committee (LREC04/015).

We have compared four groups of patients with SCA (Hb SS homozygous and Hb Sβ^0^ thalassemia hemizygous), from the UK, Nigeria, Tanzania, and the USA (Table S1). For all patients, the HbF levels (as % of total hemoglobin) were measured by HPLC (Variant II system, BioRad, Hercules, CA, USA) from samples obtained during “steady state” outpatient visits, and off hydroxyurea therapy. Common variant genotypes were generated within individual genetic studies taking place at each of the centers involved. A cohort of three hundred African British SCA patients (of West African and African-Caribbean descent (a subset was described previously [Makani et al., 2010]) was previously recruited from King's College (PI S.L. Thein), Guy's and St. Thomas’ (PI J Howard) hospitals in London, UK. Of these, a core set of 198 patients (HbS homozygous) with extensive genotype data for *HMIP-2* markers were selected for association and haplotype analysis. The Nigerian patients’ DNA samples (*n* = 192, PI A. Adekile) were from stored material from a previous study of β^S^-haplotypes involving patients from the Northern (Sokoto, Zaria, Kaduna) and Southern (Enugu, Calabar, Enugu, Benin) parts of the country (Adekile et al., 1992). Tanzanian patients from Muhimbili National Hospital, Dar-es-Salaam (*n* = 1,039, PI J. Makani) are either of Hb SS or Hb S/β^0^ genotype and have been described previously (Makani et al., 2010). Samples for 254 African American patients (HbSS and Hb S/β^0^) were collected from sources including the Cooperative Study of Sickle Cell Disease (CSSCD), Howard University and Children's Hospital Oakland. Of the 254 patients, 111 patients were recorded clinically with HbF < 3.1, whereas 133 patients had HbF > 8.6 and 10 patients with HbF in the intermediate range. We have previously (Menzel et al., 2007a) found that such subject selection can lead to an over-estimation of the frequency of the minor, HbF-boosting alleles, i.e., in extreme-phenotype (high and low HbF) European subjects we detected a frequency of 0.38 for the “C” allele of *rs9399137*, whereas in unselected Europeans the frequency was 0.26.

Data from a previous study (Thein et al., 2007), conducted in non-African populations, were included for comparison. The first is the Asian Indian Gujarati Family D, in which the *HMIP* locus was originally discovered (Thein et al., 1994; PI SL Thein), and which segregates β-thalassemia and, independently, a haplotype at the *HMIP* locus that strongly boosts HbF. The second is a cohort (*n* = 3800) of healthy British European twins (TwinsUK, PI T Spector). As HbF levels in non-anemic individuals are below the dynamic range of the HPLC detection system, the trait is represented in the twins by the fraction of red blood cells that carries HbF (“F cells”) enumerated by flow cytometry after anti-HbF staining (Thorpe et al., 1994). HbF and F cells are closely related traits that are influenced by the same set of genes (Menzel et al., 2007a; Uda et al., 2008).

### Genotyping and Sequencing

Genotypes were generated from genomic DNA isolated from peripheral white blood cells. Genotype data from previous studies were included, which had been generated as described (Menzel et al., 2007a; Thein et al., 2007; Makani et al., 2010). Additional genotyping was performed in the London lab, by the Centre National de Génotypage (Evry, France), using the Sequenom procedure, and for the TwinsUK subjects, by the Wellcome Trust Sanger Institute and National Eye Institute via NIH/CIDR. TaqMan assays (using Applied Biosystems reagents, procedures, and 3730 instrumentation) were performed in London to generate additional genotypes for the African British, Nigerian, and Tanzanian patients. Customized genotyping procedures were devised for *rs66650371*, *rs11321816*, and *rs35786788*, which are in close physical proximity to each other. Indels *rs66650371* and *rs11321816* were amplified together by PCR and then underwent fragment sizing by capillary electrophoresis on a 3130xl Genetic Analyser (Applied Biosystems, Foster City, CA, USA). For this, PCR reactions were carried out in a volume of 15 μl that contained Ampli Taq Gold (Applied Biosystems, with the buffer supplied), 2.5 mM MgCl_2_, 0.2mM each dNTP, FAM, and VIC labeled upstream primers and PIG-tailed (Brownstein et al., 1996) downstream primers under the following thermocycling conditions: 95°C for 12 min, 9 cycles at 94°C for 15 sec, 55°C for 15 sec and 72°C for 30 sec, 19 cycles of 89°C for 15 sec, 55°C for 15 sec and 72°C for 30 sec, and a final elongation at 72°C for 10 min. SNP *rs35786788* was genotyped using a SNaPshot assay (Applied Biosystems). Fragment sizing and SNaPshot reaction were both analyzed using GeneMarker software, version 1.95 from SoftGenetics (State College, PA, USA).

To investigate the critical region at the “A/a” sublocus, a 542-bp PCR amplicon (chr6:135,418,601–135,419,142; hg19) was sequenced in 18 unrelated Europeans (top panel, Fig. S1) and 15 African British patients with SCA (bottom panel, Fig. S1), all selected to be homozygous for *rs11321816* to avoid fragment shift. The fragment was first amplified from genomic DNA, using the Qiagen Muliplex PCR kit (Qiagen, Venlo, The Netherlands) with Q solution (using Qiagen recommended procedures). PCR products were purified using Wizard SV Gel and PCR clean up system and cycle-sequenced with BigDye Terminator v3.1 chemistry (Applied Biosystems). After3130XL electrophoresis, sequencing traces were inspected and scored with Sequencher 4.6 software.

Genomic DNA samples from African American patients were genotyped using Illumina HumanOmni1-Quad BeadChip System (Illumina Omni1-chip, Illumina Inc., La Jolla, CA, USA), which was designed for 76% genomic coverage for people of African ancestry. SNP genotype was called by using Illumina Genome Studio and extracted for the *HMIP-2* region for this study.

#### LD plots, phase alignment, and haplotype clades

LD between markers was estimated and plotted with Haploview 4.2 (Barrett et al., 2005). Haplotype blocks were defined using confidence intervals (minima for strong LD: 0.98 upper, 0.7 lower; upper CI maximum for strong recombination 0.9).

Phase alignment of variant alleles into haplotypes in the Gujarati family was manually inferred from segregation patterns. In sickle patients, who are unrelated, haplotypes were inferred statistically using Phase 2.1.1 (Stephens & Scheet, 2005). Haplotypes were then grouped (i.e., sorted into clades) according to the presence or absence of characteristic (“tagging”) alleles at *rs9399137* (for the “A/a” sublocus) and *rs9402686* (at the “B/b” sublocus of *HMIP-2*). Within each clade and each population/patient group, allele frequencies of the remaining genotyped variants were calculated and displayed as “sequence logos” (Schneider & Stephens, 1990), a graphical representation of the consensus and the variant alleles at each SNP position (constructed online with WebLogo 2.8.2 Crooks et al., 2004; via http://weblogo.berkeley.edu).

#### Association analysis

In the four patient cohorts, genetic association of variants with %HbF and %F cell levels (both natural-log transformed) was analyzed by multiple linear regression (SPSS, Version 12, IBM), with age and sex included as covariates. The unstandardized regression coefficient (“β”) was estimated as a measure of the effect each variant allele has on ln(HbF) levels, independent of sample sizes and allele frequencies, which differ across populations. Meta-analysis of the four groups was conducted, using a fixed-effects (inverse-variance weighted) model and included a test for heterogeneity.

In the European twins cohort, regression analysis with ln(%F cells) was carried out using the regress procedure in Stata version 10.1 after imputing missing genotypes with MACH 1.0 (Y Li and G Abecasis). Cotwin clustering was modeled by means of a modified sandwich estimator of the variance.

#### Public population genotype data

The ***1000 Genomes Project*** (Abecasis et al., 2012) is an international collaboration to generate reference genome sequences for representative human population samples, providing a comprehensive resource on human genetic variation. Phase-aligned genotype data for all variants detected during whole-genome sequencing in 1197 “first-phase” samples have been made available to researchers and are distributed over 14 populations (Table S5). Variant Call Format (VCF) files for all of the above were obtained using the “Data Slicer” tool at http://browser.1000genomes.org, specifying the input URLs as http://ftp://ftp.1000genomes.ebi.ac.uk/vol1/ftp/release/20110521/ALL.chr6.phase1_release_v3.20101123.snps_indels_svs.genotypes.vcf.gz (for the VCF file) and http://ftp://ftp.1000genomes.ebi.ac.uk/vol1/ftp/release/20110521/phase1_integrated_calls.20101123.ALL.panel (for the Sample-Population Mapping File), and the genomic region to be extracted as between chr6:135,411,228 and 135,465,800 (in hg19 coordinates). This region contains the entire *HMIP-2* locus and a 3’ adjacent 30 kb genome segment. Haplotype clades were assembled and displayed as described above for the sickle cell disease patients.

The HGDP (Conrad et al., 2006) is an international collaboration to systematically investigate the genetic history of human populations. Phase-aligned genotype data in the *HMIP-2* interval for 53 populations were accessed through the HGDP Selection Browser (Pickrell et al., 2009), a tool designed to “explore the genetic signatures of natural selection in the human genome” (http://hgdp.uchicago.edu/). Data are from 938 individuals genome-scanned on an Illumina 650K chip platform (Li et al., 2008). Populations are detailed in Table S5.

**Figure 8:**
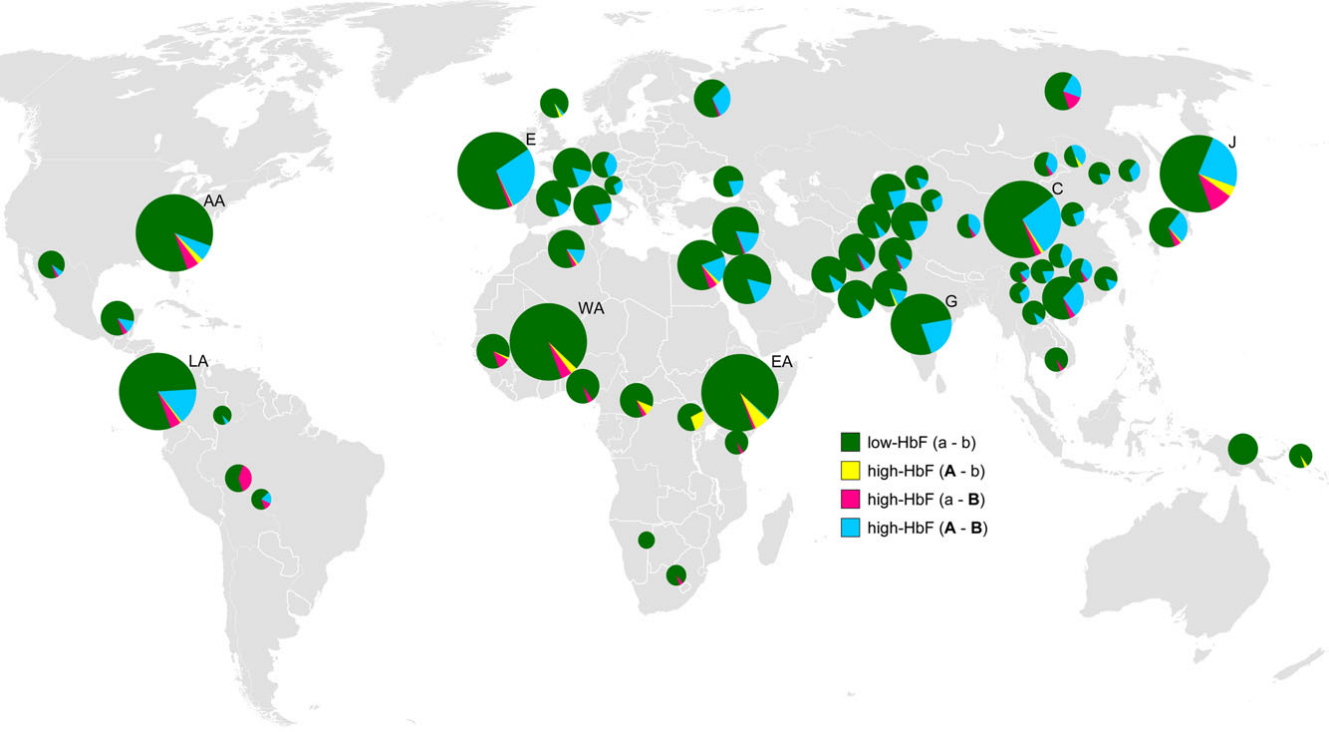
Frequency of HbF-promoting haplotype clades in 61 human populations. Phase-aligned genotypes for twelve SNP markers within the *HMIP-2* interval were available for 53 populations from the Human Genome Diversity (Pickrell et al., 2009) dataset (smaller pie charts). The presence of haplotypes was determined through tagging SNPs *rs9399137* (“A/a” sublocus of *HMIP-2*) and *rs4895441* (“B/b” sublocus). The map is showing the location of populations (Table S5) as detailed in a 2002 HGDP publication (Cann et al., 2002). The area of the chart discs is proportional to the population size. Clade frequency data are detailed in Table S5.Included are, for comparison, the six population groups from the 1000 Genomes project (as detailed in Figure7 and Table S4, chart disk size capped) and our own data for Gujarati individuals (WA: West African, i.e., Yoruba; AA: African American; EA: East African, i.e., Luhya; C: Chinese, i.e., Han; LA: Latin American, i.e., Colombian, Mexican American, Puerto Rican; E: European; G: Gujarati).Haplotypes promoting HbF persistence have a low prevalence in Sub-Saharan Africa (except in Mbuti Pygmy, who carry 26%), South East Asia and Papua New Guinea, three malaria-endemic regions, but a high frequency in East Asia and Europe.

Figure8 (world map in Robinson projection) is based on “BlankMap-World6,_compact.svg” from Wikimedia (http://commons.wikimedia.org). Haplotype frequencies (Table S5) were plotted with Inkscape 0.48 into map positions according to sampling location (Cann et al., 2002).

#### Archaic hominins

Denisova genotypes were accessed through the UCSC genome browser (track: Denisova High Coverage Sequence Reads). These originate from high-coverage genome sequence generated from a single individual (Meyer et al., 2012) and therefore would likely not capture alleles that existed in low frequency in Denisovans. Neanderthal genotypes were also retrieved through the UCSC browser (track: Neanderthal Sequence Reads, by Ed Green, UCSC) and are based on low-coverage reads (Green et al., 2010) from Neanderthal specimens from three individuals (Vi33.16, Vi33.25, and Vi33.26). Sequence from a further three individuals did not cover the critical SNPs. Additional data were available from a high-coverage genome sequence of one (“Altai Neanderthal”) individual (Prüfer et al., 2013), and was publically available (http://cdna.eva.mpg.de/neandertal/altai/AltaiNeandertal/VCF/). Thus, in total, two chromosomes were investigated for Denisovans and three, on average for Neanderthals, allowing the detection of major alleles, but making the detection of minor alleles at positions polymorphic in Neanderthals or Denisovans relatively unlikely.

#### Great Apes

Chimpanzee (Chimpanzee_Sequencing_and_Analysis_Consortium, 2005; *Pan troglodytes*), Gorilla (Scally et al., 2012; *Gorilla gorilla gorilla*), Orangutan (*Pongo pygmaeus abelii*, Washington University and Baylor College of Medicine), and Baboon (*Papio hamadryas*, Baylor College of Medicine) reference sequences were accessed through UCSC genome browser track “Multiz Alignments of 46 Vertebrates.”

## Results

### The European High-HbF Consensus Haplotype

To further characterize the European/Gujarati high-HbF genotype (or haplotype) at the *HMIP-2* locus, we sequenced the corresponding 24-kb physical region (chr6:135,411,228–135,435,501; hg19; Thein et al., 2007) in two individuals from Family D, one homozygous, through consanguinity, for the “HPFH +” (high HbF) haplotype and one compound heterozygous for “HPFH−” (low HbF) haplotypes. We detected 29 variants that are unique to the “HPFH+” sequence: 26 SNPs, two indels, and a (CA)_n_ short tandem repeat. To evaluate the biological significance of the “HPFH+” variants, we tested for association with HbF persistence (measuring “%F cells,” the proportion of red blood cells carrying HbF) in our cohort of healthy European twins. We detected eight new variants (seven SNPs and a 3-bp indel, in addition to the 12 SNPs previously described) that were strongly associated with HbF persistence (Table S2). Four markers were not or only weakly associated with the trait and for five markers assays could not be designed or failed. Exploratory sequencing of a selection of twin samples showed that two of the latter (SNP *rs9376091* and indel *rs11321816*, see also Fig. S1) were in close LD with the other associated markers, bringing the total number of strongly trait-associated variants within the *HMIP-2* interval to 22 (12 + 8 + 2; Table S2). As expected, all these variants are in close LD (Fig. S2), the minor alleles (all associated with higher HbF) forming one principal haplotype clade (23% of haplotypes) and the major alleles (associated with low HbF) forming the other principal clade (73%). For the two indels, the shorter alleles are part of the high-HbF clade, while the longer alleles reside within the low-HbF clade (detailed in Fig. S1). The composition of the (major) low-HbF clade matches the sequence of the homologous chimpanzee positions and also sequence reads available for extinct hominins (Green et al., 2010; Meyer et al., 2012; Prüfer et al., 2013; Neanderthal and Denisova; Table S3) for all trait-associated variants. Therefore this low-HbF clade was termed the “ancestral haplotype clade.”

#### HMIP-2 in individuals of African descent

**Figure 2:**
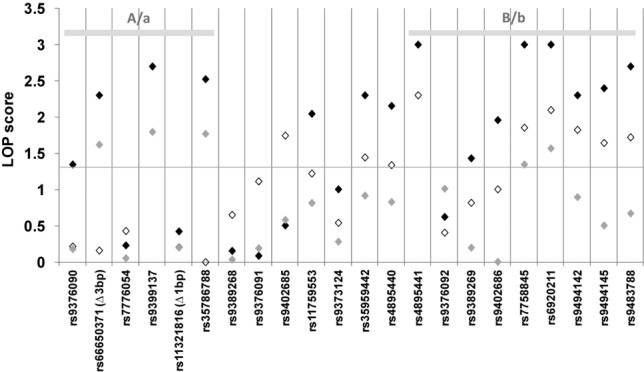
Association of variants across *HMIP-2* with HbF in African British patients with sickle cell anemia. This patient cohort (*n* = 198) contains individuals of West-African and African-Caribbean (i.e., European admixed) descent. Association between genotypes and fetal-hemoglobin persistence (%HbF, natural-log transformed) is plotted as LOP scores (−log_10_ of *P* values, black diamonds) and the threshold of nominal significance (equivalent to *P* = 0.05) is indicated as a horizontal line. The number of variants was extended to 22 (shown here in chromosomal order), all strongly associated with HbF persistence in Europeans (Table S2). The conditional independence of A/a and B/b subloci was tested by conditioning analysis on *rs9399137* (tagging A/a, open diamonds) and on *rs4895441* (the most significant marker for B/b, grey diamonds).The SNP *rs7775698*, which is part of the 3-bp in/del system *rs66650371* was also analyzed, but the C->T change had no influence on HbF levels (*P* > 0.1), which was also the case when individuals carrying the deleted allele were excluded from analysis. The length of a polymorphic microsatellite repeat present in the interval (CA_n_, chr6: 135,420,855–135,420,897) was not associated with HbF levels.

**Figure 3:**
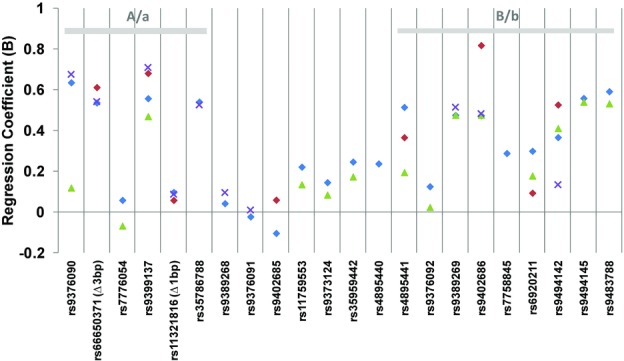
Average allelic effects of *HMIP-2* variants in ethnically diverse groups of sickle cell anemia patients. Patients are from African (Nigerian: red diamonds, Tanzanian: crosses) or African-descended (African British: blue diamonds, African American: green triangles) populations. Plotted are the estimates of the regression coefficient between genotype and trait (ln[%HbF]), with respect to the minor allele for each of the 22 European-derived markers.Direction and magnitude of the effects of individual variants are generally consistent across patient populations, as is the pattern of two spatially separated areas of association (subloci A/a and B/b).

The clinical importance of HbF persistence in the β-hemoglobin disorders has led to numerous studies investigating the European-derived association signals at *HMIP-2* in SCA patients and population cohorts of African descent (Lettre et al., 2008; Creary et al., 2009; Makani et al., 2010; Solovieff et al., 2010; Farrell et al., 2011; Bae et al., 2012). While not all of the original 12 SNPs were genotyped across all groups, several of them were found associated in each of the studies, all with the same direction of effects as in the Europeans. One notable difference to the European findings was the presence of two partially independent association signals within *HMIP-2* (Lettre et al., 2008; Galarneau et al., 2010; Makani et al., 2010), which contrasts with the single associated LD block found in Europeans. To investigate this further, we examined the 22 European-derived candidate variants for association with HbF in four groups of SCA patients of diverse African descent (Table S1), for which HbF data and genotypes have been previously generated. The most extensive SNP coverage was available in a mixed group of 198 West African and African-Caribbean (West African/European admixed) SCA patients recruited in South London (“African British patients”). HbF association of variable significance (from *P* = 0.001 to *P* = 0.045) was detected with 15 of the “European” variants, while seven variants were not associated (Fig.2; Table S2). In these African-descended individuals, the *HMIP-2* association signal appeared to be split spatially into two groups of HbF-associated markers: one group situated in the proximal (relative to the centromere, left-hand side in Fig.2) half of the block, surrounding sentinel SNP *rs9399137*, and the other, in the distal half of the block (right-hand side in Fig.2) between *rs4895441* and *rs9483788*. Each of the two groups of markers form a distinct LD block (blocks “A” and “B,” respectively; Fig. S3). SNPs from the two groups contributed separately to the overall association with HbF (Fig.2), analogous to what has been reported previously (Lettre et al., 2008; Galarneau et al., 2010; Makani et al., 2010). This pattern of association across *HMIP-2* is seen consistently in all four groups of SCA patients, which is especially evident when comparing the size of the allelic effects between markers (Fig.3; Table S2; test for heterogeneity across groups *P* = 0.77 for *rs9399137* and *P* = 0.57 for *rs9402686*), suggesting that the genetic architecture of *HMIP-2* is similar in the patient cohorts, i.e., in African British patients (West African and African Caribbean, with about 11% European admixture, based on Duffy genotype; Drasar et al., 2013), patients from Nigeria (i.e., a West African population), from Tanzania (i.e., East African) and from the United States (African American, i.e., genetically West African with 10–18% European admixture [Parra et al., 2001; Tishkoff et al., 2009;]). Consequently, we propose the existence of two subloci within *HMIP-2*: “A/a” (at the 5’ end) and “B/b” (at the 3’ end), each possessing a high-HbF form (alleles “A” and “B”) or a low-HbF form (alleles “a” and “b”).

**Figure 4:**
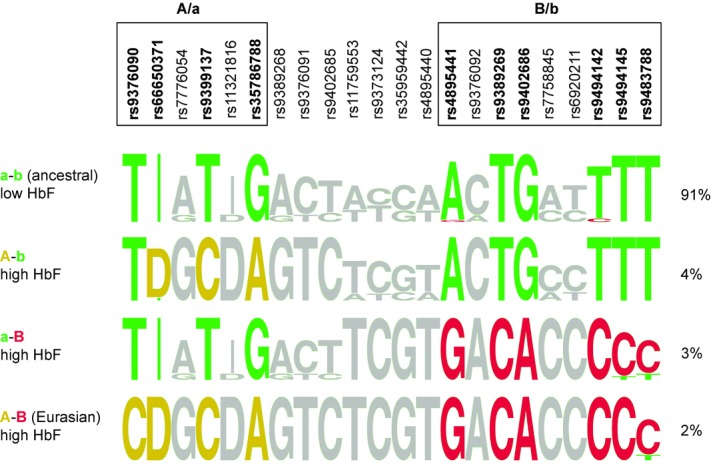
Consensus composition of the four principal haplotype clades at *HMIP-2* in African British patients. Each row depicts one of the clades (displayed as a sequence logo [Schneider & Stephens, 1990]), with the consensus allele(s) shown at each variant position. Clades were defined through *rs9399137* (tagging A/a) and *rs9402686* (tagging B/b). The height of the letter or stack indicates the degree of consensus and the relative height of letters in a stack shows the relative frequency of alternative alleles within the clade. Alleles with significant effect size (β > 0.3; Fig.3) have been colored: those associated with increased HbF levels are either orange (“A”) or red (“B”) and those associated with decreased HbF levels are green. Variants with little or no effect on HbF (<0.3; Fig.3) are shown in grey. “I” and “D” stand for insertion (“in”) or deletion (“del”) alleles, respectively.The variants representing the “A” and “B” high-HbF alleles have a high degree of consensus and specificity for their respective clades. African-type high-HbF clades (“A–b” and “a–B”) contain either high-HbF allele (“A” or “B”) separately. In the Eurasian clade, which is present through European admixture, both alleles are combined to form a single haplotype.

**Figure 5:**
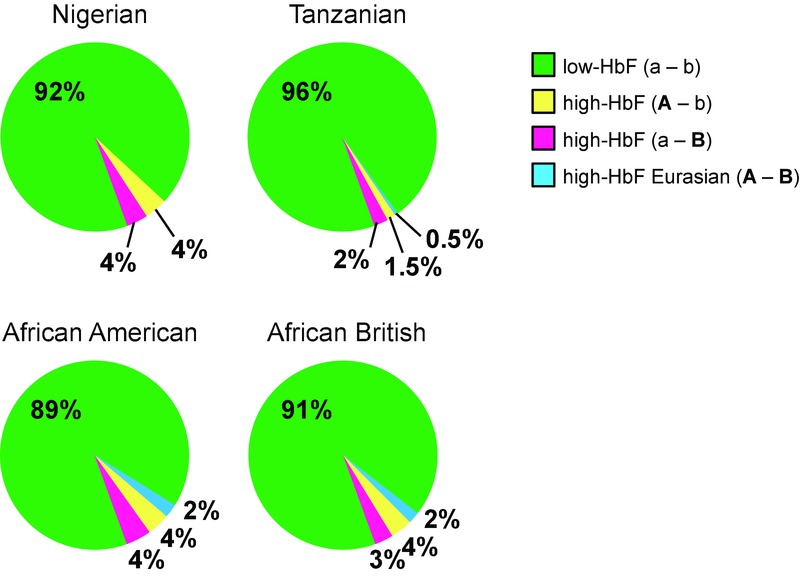
Frequency of the four *HMIP-2* haplotype clades in four African-descended patient populations. Haplotypes were grouped into clades according to alleles present for the tagging SNPs (*rs9399137* for “A” and *rs9402686* for “B”). The consensus sequence for the clades is shown in Figure4.

A mathematical reconstruction of the phase relationship of the 22 variants in the African British patients (Fig.4) reveals the haplotype architecture underlying the trait-association and LD findings. The 10 variants with the greatest allelic impact on HbF values (β > 0.3) appear to form four distinct high-consensus haplotype clades (Fig.4). The most prevalent clade (“a–b,” frequency 91%) is characterized by the presence of the low-HbF associated (ancestral) alleles at each of the 10 positions, analogous to the European ancestral haplotype clade. The remaining 12 positions (nonassociated variants) are more variable in the African version of this clade. A second, small group of haplotypes (“A–B,” 2%) shows the converse situation: high-HbF alleles for all of the ten strongly associated positions. This clade is, across all 22 variants, identical to the European high-HbF clade and also includes the European-ancestry informative allele, *rs9376090*-“C,” which is consistent with the hypothesis that these haplotypes joined the patients’ gene pool through European admixture (11% admixture from a European population carrying this haplotype at 22% would predict a frequency of 2.4%). The majority of HbF-increasing alleles at the 10 critical positions reside in two haplotype clades that contrast with the European-type (“all-or-nothing”) situation. One of these clades (“A–b,” 4% frequency) contains the high-HbF alleles at three positions within the “A/a” sublocus, but not at the “B/b” sublocus and the other clade (“a–B,” 3% frequency) is characterized by a strong consensus for the high-HbF alleles at six positions within the “B/b” sublocus only (Fig.4). Thus, HbF-increasing alleles exist within two distinct clades of haplotypes (“A–b” and “a–B”) on African chromosomes, while on European chromosomes they form a joint “A–B’ (tandem) high-HbF clade. We detected three of these four haplotype clades, defined by the ten trait-associated variants, in all four patient groups in this study (West African, East African, African American, and African British); the “European-type” clade (“A–B”), was absent from the Nigerian patients (Figs 4 and 5).

The four high-HbF associated alleles within “A” are *rs9376090*-“C” (restricted to European chromosomes), *rs66650371*-“del,” *rs9399137*-“C” and *rs35786788-“A*.” Thus the haplotype signature “del-C-A” tags the presence of a functional, HbF-promoting “allele A” at this sublocus in European, North Indian (Gujarati), and diverse African populations. It has previously been suggested that *rs66650371*-“del” itself might be biologically effective and responsible for HbF association at *HMIP-2* (Farrell et al., 2011), even though direct biological proof has remained elusive. High-HbF associated variants within “B” are *rs4895441*-“G,” *rs9389269*-“C,” *rs9402686*-“A,” *rs9494142*-“C,” *rs9494145*-“C” and *9483788*-“C” (Fig.4). Therefore, the haplotype signature “G-C-A-C-C-C” indicates the presence of a so-far unidentified functional “allele B” and might serve as its proxy in studies in a wide range of human populations.

#### Effects on HbF persistence

The three high-HbF clades (A–b, a–B, and A–B) seem to have similar effects on HbF persistence in SCA patients, with similar regression coefficients (i.e., average per-allele effects of the minor allele on ln[%HbF]) for tag SNPs representing the subloci (Fig.3 and Table S2), e.g., +0.62 for *rs9399137-C* (tagging “A–b” and “A–B”), +0.51 for *rs9402686-A* (tagging “a–B” and “A–B”) and +0.58 for *rs9376090-C* (tagging “A–B” only). Thus the European clade, containing “A” as well as “B” alleles, appeared to have an effect no larger than the two alleles on their own, though variability across groups was considerable (Fig.3; Table S2). We therefore investigated these clades in the European cohort (*n* = 3800), where the frequency of “A–B” is 23%, and where there are small numbers (∼0.6% each) of chromosomes carrying only either “A” or “B.”

**Figure 6:**
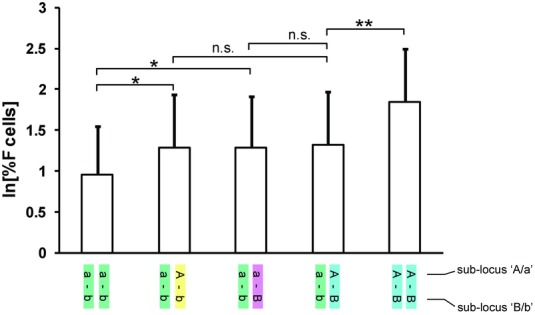
Fetal haemoglobin persistence (%F cells) in Europeans stratified by *HMIP-2* genotype. The European cohort was chosen, because of its size (*n* = 3800), to compare genotypic trait values (mean + SD) for individuals carrying different haplotype combinations.Carrying a single HbF-promoting allele (second and third column) significantly boosts HbF persistence, compared to having none (first column). The effects of “A” and “B” alleles appear similar. Carrying a “double-hit” chromosome (“A–B” haplotype) does not further increase trait values when present in heterozygous form. In comparison, homozygotes have significantly increased F cell levels. Genotypic values for HbF persistence in the twins were measured as “% F cells,” the proportion of red blood cells carrying HbF. This is equivalent to “% HbF” but better suited to nonanemic subjects, where %HbF values are below the dynamic range for the standard HPLC detection method.*P < 0.005; **P < 0.0001; n.s., not significant.A one-tailed *t*-test was used for comparing the genotype groups, i.e., to confirm/reject the findings in the patients, i.e., an HbF-increasing effect of the “A” and “B” containing haplotypes.

Trait values for individuals carrying each of these haplotypes in various genotype combinations are shown in Figure6. The data mirror results from the patients: individuals carrying “A–b,” “a–B,” or the “A–B” combined clade show similar effects on HbF persistence, which suggests a “dominant in cis” model for the interaction of the two subloci, where “A” or “B” produce the full phenotype independent of which allele is present at the other sublocus. However, we were unable to formally reject the possibility that “A” and “B” show additive effects in cis (*P* = 0.15); access to larger datasets or populations with higher frequencies of “A–b” and “a–B” are required to sufficiently power this test. Homozygotes for “A–B” showed significantly increased trait values (Fig.6) compared to heterozygotes, suggesting an “additive in trans” model for the locus. Again, the study of further populations might provide additional evidence, e.g., higher frequencies of “A–b” and “a–B” haplotypes might allow the assessment of their homozygotes and “A–b”/“a–B” compound heterozygotes.

#### Global prevalence of HbF-promoting haplotypes

**Figure 7:**
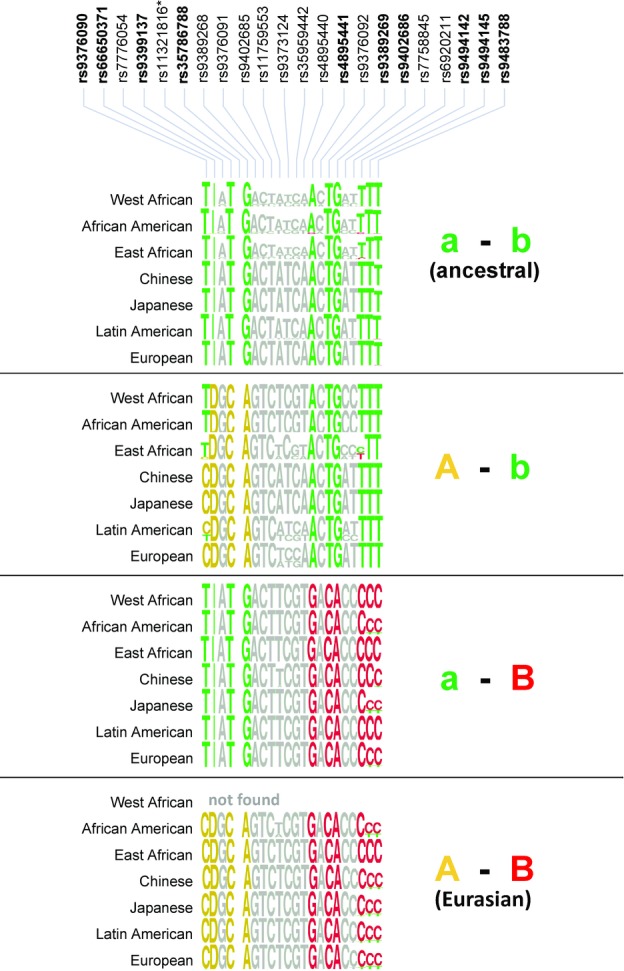
Consensus composition of the four *HMIP-2* haplotype clades in major population groups. The consensus composition (sequence logos [Schneider & Stephens, 1990]) for the *HMIP-2* haplotype clades was assembled from phased genotyped data provided by the 1000 Genomes Project (Abecasis et al., 2010). The fourteen reference populations were pooled into seven groups to increase sample numbers. HbF-associated variants (colored) and clade definition are the same as in Figure4.These data show that the identity and composition of *HMIP-2* haplotype clades across global populations is consistent and matches those present in healthy Europeans and African-descended SCA patients (Fig.4): only two types of HbF-increasing haplotype signatures are present, those of the “A” and of the “B” type. Clades are either ancestral (“a–b,” typical for a low HbF situation), or “A–b,” “a–B”, and “A–B” (all predicted to carry high-HbF alleles). Allele frequency data underlying these plots are detailed in Table S4.*rs113211816 data were not available.

The existence of common haplotype signatures for HbF-promoting alleles at *HMIP-2-A/a* and *HMIP-2-B/b* subloci across very different population groups (European/North Indian and Sub-Saharan African) suggests that the various instances of each, “A” and “B” allele, are derived from common ancestors and that they might be a general feature of human populations. To systematically investigate the presence of such alleles in populations across the globe, we looked for the presence of their characteristic haplotype signatures in public data from the 1000 Genomes Project (Abecasis et al., 2010), a repository of full genome sequence for representative human populations. For this, we retrieved phase-aligned genotype data (statistically-derived most-likely haplotypes) for 325 variants detected in the *HMIP-2* interval for 1197 individuals in fourteen populations (Phase 1) from the project's public online data repository. This included data on 21 of the 22 variants associated with HbF variants in Europeans. When grouping the haplotypes into clades based on tag SNPs *rs9399137* (representing “A/a”) and *rs9402686* (representing “B/b”), the remaining seven HbF-associated variants displayed a high-consensus pattern within these clades in all populations, analogous to what had been observed in the sickle patients. That is, in each of the populations sampled, haplotypes carrying the low-HbF variant for both tag SNPs display a high consensus for having low-HbF alleles at the remaining seven positions that define both subloci (ancestral clade; Fig.7; Table S4), haplotypes carrying the high-HbF tag allele at one of the subloci, but not at the other, again display a high consensus for the remaining high-HbF alleles at the same sublocus and for low-HbF alleles at the other one (“A–b” and “a–B” clades), and finally, haplotypes carrying high-HbF alleles for both tag SNPs carry high-HbF variant for all nine critical positions (“A–B” clade). The latter, combined, “A–B” high-HbF clade, is absent from the West African subjects, but is prevalent not only in the European populations (27%) and our own Gujarati dataset (22%), but also found at high frequency in Chinese (Han, 26%) and Japanese populations (24%), suggesting that this “Eurasian clade” was a distinguishing feature of the founder population of anatomically modern humans originally expanding into these regions. Mirroring the situation in British African and African American sickle patients, the presence of Eurasian clade in the African American population sample (at 6.6% frequency) is likely due to European admixture.

The three Latin American populations sampled in the 1000 Genomes project (Mexican American from Los Angeles, Colombian, Puerto Rican) exhibit a high frequency of *HMIP-2* haplotypes. These populations are admixed between Europeans and Amerindians (Parra et al., 2004), where *HMIP-2* haplotypes are similarly prevalent (see below). Of all groups studied in Phase 1 of the 1000 Genomes project, the Japanese population had the highest frequency (38%) of HbF-increasing haplotypes, contributed from the Eurasian and “a–B”-type clades.

The detection of the same high-consensus haplotypes in global populations as the ones we found associated with HbF persistence in our phenotyped cohorts makes it very likely that they signal the presence of HbF promoting alleles “A” and “B” and their respective unidentified biologically functional components. Thus we feel confident to evaluate the presence of such alleles in populations where erythroid traits have not been measured, analogous to the process of imputation of ungenotyped DNA variants using surrounding markers and the knowledge of their LD relationship. Furthermore, the strong LD between the characteristic variants within each sublocus allows us to extend such studies into sparser datasets, where full haplotype signatures cannot be ascertained, but where individual component SNPs can be used to tag the presence of alleles “A” and “B”. Such a dataset, providing us with an especially wide geographical and ethnic spread, was generated by the HGDP (Pickrell et al., 2009), a study of 53 human populations genome-scanned with >600,000 SNP markers. We accessed and analyzed public HGDP genotype data (in phase-aligned format), which included three of the 10 SNPs defining “A” and “B” haplotype signatures and we selected *rs9399137*, tagging “A/a,” and *rs4895441*, tagging “B/b” (similar to *rs9402686*; Fig.7), to mark the presence of the four haplotype clades. Their frequencies in each population sample, plotted to the geographical sampling position, are shown in Figure8.

HGDP data (Fig.8; Table S5) confirm the initial observation from the 1000 Genomes data: HbF-increasing alleles are generally infrequent in Sub-Saharan Africa, which lacks the combined “A–B” Eurasian clade. Conversely, this clade is common to European, Middle Eastern, Middle, and South Asian as well as East Asian populations. “A–b” and “a–B”-type high-HbF haplotypes exist at generally low frequencies in Africa with the exception of the San population, where they are absent, and two Pygmy populations, where “A–b” is unusually prevalent. A pattern of Eurasian together with “a–B” type haplotypes is common to a group of North East Asian (such as Yakut and Japanese) and Amerindian (such as Pima and Maya) populations. HbF-increasing haplotypes also appear to be rare in Cambodia (South-East Asia), New Guinea, and Bougainville (both Oceania), reinforcing our hypothesis that such clades might have a low frequency in Malaria-endemic regions (Hay & Snow, 2006).

## Discussion

*HMIP-2*, a QTL affecting fetal-hemoglobin persistence and other erythroid traits, is located within the major distal enhancer for *MYB* (Stadhouders et al., 2012; Stadhouders et al., 2014), which encodes cMYB, one of the key transcription factors regulating erythropoiesis and hematopoiesis (Mucenski et al., 1991). We have tracked the presence of two alleles affecting HbF persistence at *HMIP-2* in patients with SCA and in global human populations through characteristic SNP haplotype signatures. These alleles, “A” and “B,” which have similar effects on HbF persistence, are located in different regions of the locus. Principal haplotype clades, at the site of these alleles, can be of three types: those associated with low HbF levels (“a–b”), those leading to higher HbF containing either “A” or “B’ (clades “A–b” and “a–B”), and those containing both high-HbF alleles (“A–B”).

Physically, the “A/a” and “B/b” subloci map to distinct regulatory elements of the *MYB* enhancer (Stadhouders et al., 2014). These elements are defined through binding of the essential erythroid LDB1 transcription factor complex (Soler et al., 2010; LDB1, GATA1, TAL1, ETO2, KLF1) and physically interact with the *MYB* promoter through chromatin looping, forming a three-dimensional active chromatin hub that promotes MYB transcription (Stadhouders et al., 2012). The human enhancer contains seven such elements, four of which form a highly conserved and regulatory active “core” (LDB1 sites −87, −84, −71, and −63, relative to the MYB transcription start site). The haplotype signature for the “A” high-HbF allele occupies a 542-bp DNA fragment (Fig. S1) that largely overlaps the −84 LDB1 site. *rs66650371*-“del,” a 3-bp deletion, is one of the three variants belonging to this signature and has been proposed as a potential biologically significant allele, directly causing part of the local trait association (Farrell et al., 2011; Stadhouders et al., 2014). Variants belonging to the “B” high-HbF allele occupy a ∼9 kb fragment between *rs4895441* at 135,426,573 and *rs9483788* at 135,435,501, which includes the critical LDB1 site −71. Thus, while the “A” and “B” alleles are separated by >7 kb of sequence, important factor binding motifs within each are likely to be physically close when forming the active chromatin hub in erythroid progenitors. We suggest “A/a” and “B/b” might affect assembly of the same transactivation complex, a situation that would explain our observation of similar effects of “A–b,” “a–B,” and “A–B” haplotypes (Fig.6). Genetically, no obvious candidate for a biologically causative variant has yet emerged for the “B” high-HbF allele. None of its six signature SNPs appears to show consistently strong effects across all populations studied. Additional variants at “B/b” with strong trait association (e.g., small deletions) might be absent from public sequence data due to uncertainties in allele calling, similar to the AAAC/AACCC length polymorphism *rs11321816* (not HbF associated), which is missing from the 1000 Genomes dataset. Beyond “A” and “B”, common alleles with comparable impact on erythroid traits are unlikely to exist at this locus, since association studies with haematological traits have consistently identified variants that belong to the “A–b,” “a–B,” or “A–B” clades, including African (Makani et al., 2010), African American (Lettre et al., 2008; Solovieff et al., 2010; Farrell et al., 2011), African Caribbean (Creary et al., 2009), European (Menzel et al., 2007a; 2007b; Uda et al., 2008; Soranzo et al., 2009b; Ganesh et al., 2009; Nalls et al., 2011; van der Harst et al., 2012), Thai (Nuinoon et al., 2010), Japanese (Kamatani et al., 2010), and Chinese (Gibney et al., 2008; So et al., 2008; Farrell et al., 2011) populations, with all top association signals being components of “A” and “B” signatures and restricted to the *HMIP-2* interval. A group of strongly HbF-associated variants detected in a Southern Chinese population (Farrell et al., 2011) is largely identical with the 22 variants we found in Europeans.

The functionally significant MYB enhancer polymorphisms at *HMIP-2* are likely to have contributed to the diversity and robustness of human populations for a very long time. The “A–B” (“Eurasian-type”) high-HbF haplotype, which is common to most populations outside of Africa, is likely to have been prevalent in the founder population that started populating the rest of the world during the last interglacial period >125,000 years ago (Armitage et al., 2011). The origin of “A–B” therefore lies most likely in East Africa, near the waypoint for the out-of-Africa migration of modern humans (Tishkoff et al., 2009). The Kenyan Luhya population sample (*n* = 97) of the 1000 Genomes project harbors small amounts of European-like haplotypes: a single “A–B” haplotype and two instances of an “A–b” haplotype with European-like features (*rs9376090*-C), which could either point towards the possible source of Eurasian haplotypes from within East Africa or, alternatively, be due to back-migration from Eurasia (Pickrell et al., 2014). The Nigerian Yoruba sample (*n* = 88) has no such European-like features. High-HbF haplotypes belonging to “A–b” and “a–B” clades would have existed in Sub-Saharan Africa long before the expansion out of Africa, given their wide, albeit low frequency, distribution on this continent. Presently, it cannot be excluded that these polymorphisms might have predated anatomically modern humans. While the presence of a single read containing an allele belonging to “B” in the Neanderthal sequence pool (Table S3) could be an artifact, it is the prevalence gradient of “a–B” haplotypes (red in Fig.8) across the Asian-European landmass (extending to the Americas), mirroring an East–West gradient for Neanderthal (Wall et al., 2013) and Denisovan (Skoglund & Jakobsson, 2011) ancestry in extant human populations, that might lead to the speculation that such alleles could have existed in East Asia before the arrival of modern humans.

Powerful selection pressure exerted by malaria has shaped the distribution of erythroid trait variants across the world, with the largest impact in tropical regions, which have consistently supported malaria parasites and their insect vectors throughout primate evolution (Carter & Mendis, 2002). Similarly, the overall lower frequency of “A” and “B” alleles in Sub-Saharan Africa, South-East Asia, and Oceania could be due to negative selection against homozygotes or compound heterozygotes by malaria parasites, especially *Plasmodium vivax*, which could have been present in Africa since the divergence of human and chimpanzee lines (Carter & Mendis, 2002). *HMIP-2*-controlled traits, such as red cell number, size, and hemoglobin content, in addition to F cell increase and HbF persistence, are thought to arise through the intervention of cMYB in the kinetics of erythropoiesis (Thein et al., 2009). Such changes might influence red cell invasion (Pasvol et al., 1980), parasite density (Louicharoen et al., 2009), or survivability (Villeval et al., 1990) of plasmodium infection, or interfere with other protective alleles, analogous to what has been suggested for alpha thalassemia and sickle cell mutations (Williams et al., 2005). Positive selection pressures might predominate elsewhere, possibly reflecting specific demands on red blood cell production, such as altitude adaptation (Huerta-Sanchez et al., 2013). Nutritional factors, such as the availability of iron or vitamins, can also affect erythropoiesis (Hoffbrand et al., 2011) and might require long-term adaptation.

We have presented evidence that two functionally similar but evolutionarily distinct enhancer polymorphisms affecting erythroid traits are present in most human populations. Their wide distribution will aid further mapping efforts to identify their biologically functional constituents. We propose that the comparison of haplotypes harboring critical *HMIP-2* variants between populations will be a useful tool in tracing human migration and assimilation through much of our evolutionary history. During these events, environmental challenges might have led to different demands on the generation of red blood cells. Exploring these processes and their genetic consequences will contribute to our understanding of human erythroid biology.

## Supporting Information

**Figure S1** Detailed haplotype composition of the core of the “A/a” sublocus in individuals of European and African descent. **Figure S2** Linkage disequilibrium plot for 21 variants across *HMIP-2* in 2183 healthy Europeans. **Figure S3** Linkage disequilibrium plot for 20 variants across *HMIP-2* in 198 African British patients with sickle cell anemia. **Table S1** Groups of patients with sickle cell anemia investigated in this study. **Table S2** Association of candidate variants with fetal-hemoglobin persistence in Europeans and in African-descended patients with sickle cell anemia. **Table S3** Genotypes for HbF-associated variants at *HMIP-2* in archaic hominins and in great apes. **Table S4** Frequency of SNP alleles associated with HbF persistence within haplotype clades “a–b,” “A–b,” ‘a–B,” and “A–B” in seven population groups from the 1000 Genomes project. **Table S5** Frequencies of *HMIP-2* haplotype clades in human reference populations.
