# Supplementary material for: Global Genetic Architecture of an Erythroid Quantitative Trait Locus, HMIP-2
Source: Ann Hum Genet. 2014 Jul 29;78(6):434–51. doi: 10.1111/ahg.12077 (PMC4303951; doi:10.1111/ahg.12077)
Supplement: Supplementary file 1 — Figure S1 Detailed haplotype composition of the core of the “A/a” sublocus in individuals of European and African descent. Figure S2 Linkage disequilibrium plot for 21 variants across HMIP-2 in 2183 healthy Europeans. Figure S3 Linkage disequilibrium plot for 20 variants across HMIP-2 in 198 African British patients with sickle cell anemia. Table S1 Groups of patients with sickle cell anemia investigated in this study. Table S2 Association of candidate variants with fetal-hemoglobin persistence in Europeans and in African-descended patients with sickle cell anemia. Table S3 Genotypes for HbF-associated variants at HMIP-2 in archaic hominins and in great apes. Table S4 Frequency of SNP alleles associated with HbF persistence within haplotype clades “a–b,” “A–b,” ‘a–B,” and “A–B” in seven population groups from the 1000 Genomes project. Table S5 Frequencies of HMIP-2 haplotype clades in human reference populations. [file ahg0078-0434-sd1.zip › ahg12077-sup-0001-FigureS1.pdf]

HAPLOTYPE		29 bp (non-polymorphic)	non-polymorphic	**rs6650371 (TAY/---)**	rs7775698 (TAC/TAT)	non-polymorphic	25 bp (non-polymorphic)	rs61028892 (G/C)	251 bp (non-polymorphic)	rs7776054 (A/G)	71 bp (non-polymorphic)	rs7776196(A/G)	29 bp (non-polymorphic)	**rs9399137(T/C)**	17 bp (non-polymorphic)	rs11321816(CC/A-)	non-polymorphic	**rs35786788(G/A)**	100 bp (non-polymorphic)	population frequency
**European non-anemic cohort**																				
**A**		—	TGT	---		TAT	—	G	—	G	—	A	—	C	—	AAA-C	T	A	—	23%
**a**	**a_1**	—	TGT	TAC		TAT	—	G	—	A	—	A	—	T	—	AACCC	T	G	—	77%
**British patients with sickle cell anemia (African chromosomes)**																				
**A**		—	TGT	---		TAT	—	G	—	G	—	A	—	C	—	AAA-C	T	A	—	4%
**a**	**a_1**	—	TGT	TAC		TAT	—	G*	—	A	—	A	—	T	—	AACCC	T	G	—	78%
	**a_2**	—	TGT	TAT		TAT	—	G	—	G**	—	G	—	T	—	AAA-C	T	G	—	18%

Figure S1: Detailed haplotype composition of the core of the 'A/a' sub-locus in individuals of European and African descent.
