# Supplementary material for: Global Genetic Architecture of an Erythroid Quantitative Trait Locus, HMIP-2
Source: Ann Hum Genet. 2014 Jul 29;78(6):434–51. doi: 10.1111/ahg.12077 (PMC4303951; doi:10.1111/ahg.12077)
Supplement: Supplementary file 1 — Figure S1 Detailed haplotype composition of the core of the “A/a” sublocus in individuals of European and African descent. Figure S2 Linkage disequilibrium plot for 21 variants across HMIP-2 in 2183 healthy Europeans. Figure S3 Linkage disequilibrium plot for 20 variants across HMIP-2 in 198 African British patients with sickle cell anemia. Table S1 Groups of patients with sickle cell anemia investigated in this study. Table S2 Association of candidate variants with fetal-hemoglobin persistence in Europeans and in African-descended patients with sickle cell anemia. Table S3 Genotypes for HbF-associated variants at HMIP-2 in archaic hominins and in great apes. Table S4 Frequency of SNP alleles associated with HbF persistence within haplotype clades “a–b,” “A–b,” ‘a–B,” and “A–B” in seven population groups from the 1000 Genomes project. Table S5 Frequencies of HMIP-2 haplotype clades in human reference populations. [file ahg0078-0434-sd1.zip › ahg12077-sup-0009-SuppMat.docx]

**SUPPORTING INFORMATION**

**Global genetic architecture of an erythroid quantitative trait locus, *HMIP-2***

**Figure S1: Detailed haplotype composition of the core of the ‘A/a’ sub-locus in individuals of European and African descent.**

Sequence data shown are of the core 542-bp fragment (chr6:135,418,601-135,419,142; hg19) that contains the three HbF signature markers *rs6650371*, *rs9399137* and the *rs35786788*. Across the 18 Europeans and 15 West-African descended individuals sequenced, the ‘A’ clade (high-HbF, in Europeans present in ‘A – B’ constellation and in Africans in ‘A – b’ constellation) consists of a single haplotype, which is identical in both populations. The ancestral ‘a’ clade (low HbF) appears more diverse, especially on West African chromosomes, where SNPs without HbF effect (p = 0.285) split the clade into two major haplotypes, ‘a_1_’ and ‘a_2_’. Nucleotides are shown for variant positions only, with a few non-polymorphic nucleotides for reference.

The haplotype situation presented here is in agreement with results described by Farrell et al. (Farrell *et al.*, 2011) for their African-American cohort.

* A 'C' allele for was encountered once in this position.

** An additional G/C polymorphism 4 bp 3’ of *rs7776054* was encountered once within the 'a_2_' haplotype.

**Figure S2: Linkage disequilibrium plot for 21 variants across HMIP-2 in 2183 healthy Europeans.**

HbF-associated variants across the region form a major haplotype block, which includes sub-locus A/a (*rs66650371* to *rs35786788*) and sub-locus B/b (*rs4895441*, *rs9389269*, *rs9402686*) are within Block B. The remaining four variants (also part of sub-locus B/b, *rs6920211* to *rs9483788*) are also in close LD with this block.

The plot was created with Haploview 4.2 (Barrett *et al.*, 2005). In the colouring scheme used, shades of pink/red indicate the degree of LD (D’) when the LOD is > 2, white indicates no LD (LOD<2, D’<1), blue marks a D’ of 1, but LOD<2. Numbers within the squares show pairwise r^2^.

**Figure S3: Linkage disequilibrium plot for 20 variants across *HMIP-2* in 198 African British patients with sickle cell anaemia.**

Variant of the two groups of HbF-associated variants reside in distinct LD blocks. Variants at sub-locus A/a (*rs66650371*, *rs9399137* and *rs35786788*) are within Block A in above plot and SNPs for sub-locus B/b (*rs4895441*, *rs9389269*, *rs9402686*) are within Block B. Additional HbF-associated variants in LD with Block B are *rs9494142* and *9483788*.

*rs9376090*, in LD with markers across the entire interval, is an ancestry-informative marker tagging Eurasian (combined A – B) haplotypes, which are present through admixture.

The plot was created with Haploview 4.2 (Barrett *et al.*, 2005). Shades of pink/red indicate the degree of LD (D’) when the LOD is > 2, white indicates no LD (LOD<2, D’<1), blue marks a D’ of 1, but LOD<2. Numbers within the squares show pairwise r^2^.

**Table S1: Groups of patients with sickle cell anaemia investigated in this study.**

*Muhimbili Sickle Cohort

**As reported elsewhere for the general African American population (Tishkoff *et al.*, 2009, Parra *et al.*, 2001)

**Table S2: Association of candidate variants with fetal-haemoglobin persistence in Europeans and in African-descended patients with sickle cell anaemia.**

Fetal-haemoglobin persistence was measured by flow cytometry (proportion of red blood cells carrying fetal haemoglobin) in non-anaemic Europeans and by HPLC (proportion of HbF in total haemoglobin) in patients in order to apply methods with a dynamic range matching the trait levels in the respective groups.

* *rs11321816* and *rs9376091* were not genotyped but sequencing experiments suggested a close LD with *rs9399137* and *rs9389268*, respectively.

Four further variants were found to not or only weakly associated with HbF persistence (*rs2210366*, *rs6930223*, *rs55936352* and *rs7766963*) and for three operational assays were not available (the (Ca)_n_ STR *MSc*, *rs11969203* and *rs34164109*).

**Table S3: Genotypes for HbF-associated variants at HMIP in archaic hominins and in great apes.**

Shown are genotype data at four HbF-associated positions at HMIP-2 A/a and six such variants at HMIP-2 B/b for two groups of archaic hominins (Denisova and Neanderthal) and for the four great ape species. Denisovan genotypes are from a single individual (derived from high coverage sequencing), while Neanderthal data are from three low coverage genomes, sampling, on average, a single chromosome at each site and from the recent high-quality whole-genome sequence generated from the Altai Neanderthal individual. Since hominin data are still sparse, undetected additional alleles for these positions might have existed in both groups’ populations.

The sequence at the positions shown (i.e. those consistently associated with HbF in extant humans) mostly matches the human low-HbF alleles, indicating that these alleles and the haplotypes they exist in represent the ancestral situation (therefore termed ‘ancestral haplotype clade’). In Neanderthal individual Vi33.26, a single read contained a high-HbF allele (at *rs9402686*), raising the possibility that this individual might have been heterozygous for an HbF-raising variant similar to the 2B allele found in extant humans. It seems at least equally likely though that this finding has arisen due to a comparatively low quality of this read (i.e., the quality score is 40% throughout, and a variant nucleotide was also found in the immediately neighbouring base position).

* 1 of 29 reads shows a 'G' in this position

** 1 of 30 reads shows an 'A' in this position

*** The 'I' allele for this marker contains an additional SNP, *rs7775698* C/T, which is not HbF-associated and not a signature SNP for the clades. In general, positions not associated with HbF are not shown.

**Table S4: Frequency of SNP alleles associated with HbF persistence within haplotype clades ‘a – b’, ‘A – b’, ‘a – B’ and ‘A – B’ in seven population groups from the 1000 Genomes project.**

For each of the 22 variants associated with HbF persistence in Europeans, the frequency of the HbF-promoting allele is given. The data presented in this table are the same as those underlying Figure 7.

**Table S5: Frequencies of *HMIP-2* haplotype clades in human reference populations.**

Frequencies for haplotype clades (low HbF ancestral ‘a-b’, high-HbF Eurasian ‘A-B’, high-HbF ‘A-b’ and high-HbF ‘a-B’) are based on the presence of tagging SNP alleles as identified from public datasets (references). These frequencies are plotted in Figure 8.

Data from a North Indian (Gujarati) population was added from our own data, represented by unique (non-identical by descent) haplotypes segregating in Family D.
